# Trends in Psychological Distress Among Adults in England, 2020-2022

**DOI:** 10.1001/jamanetworkopen.2023.21959

**Published:** 2023-07-06

**Authors:** Sarah E. Jackson, Jamie Brown, Lion Shahab, Ann McNeill, Marcus R. Munafò, Leonie Brose

**Affiliations:** 1 Department of Behavioural Science and Health, University College London, London, United Kingdom; 2 SPECTRUM Consortium, United Kingdom; 3 Addictions Department, Institute of Psychiatry, Psychology and Neuroscience, King’s College London, London, United Kingdom; 4 MRC Integrative Epidemiology Unit, School of Psychological Science, University of Bristol, Bristol, United Kingdom

## Abstract

**Importance:**

In the last 3 years, people in England have lived through a pandemic and cost-of-living and health care crises, all of which may have contributed to worsening mental health in the population.

**Objective:**

To estimate trends in psychological distress among adults over this period and to examine differences by key potential moderators.

**Design, Setting, and Participants:**

A monthly cross-sectional, nationally representative household survey of adults aged 18 years or older was conducted in England between April 2020 and December 2022.

**Main Outcomes and Measures:**

Past-month distress was assessed with the Kessler Psychological Distress Scale. Time trends in any distress (moderate to severe, scores ≥5) and severe distress (scores ≥13) were modeled, and interactions with age, gender, occupational social grade, children in the household, smoking status, and drinking risk status were tested.

**Results:**

Data were collected from 51 861 adults (weighted mean [SD] age, 48.6 [18.5] years; 26 609 women [51.3%]). There was little overall change in the proportion of respondents reporting any distress (from 34.5% to 32.0%; prevalence ratio [PR], 0.93; 95% CI, 0.87-0.99), but the proportion reporting severe distress increased by 46%, from 5.7% to 8.3% (PR, 1.46; 95% CI, 1.21-1.76). Although trends differed by sociodemographic characteristics, smoking, and drinking, the increase in severe distress was observed across all subgroups (with PR estimates ranging from 1.17 to 2.16), with the exception of those aged 65 years and older (PR, 0.79; 95% CI, 0.43-1.38); the increase was particularly pronounced since late 2021 among those younger than 25 years (increasing from 13.6% in December 2021 to 20.2% in December 2022).

**Conclusions and Relevance:**

In this survey study of adults in England, the proportion reporting any psychological distress was similar in December 2022 to that in April 2020 (an extremely difficult and uncertain moment of the COVID-19 pandemic), but the proportion reporting severe distress was 46% higher. These findings provide evidence of a growing mental health crisis in England and underscore an urgent need to address its cause and to adequately fund mental health services.

## Key Points

**Question:**

How has the prevalence of psychological distress in the adult population of England changed since 2020?

**Findings:**

This survey study of 51 861 adults found that the proportion reporting severe levels of distress increased steadily by 46%, from an already elevated baseline, since the start of the pandemic. This increase in severe distress occurred across all population subgroups, with the exception of older adults (aged ≥65 years), and was most pronounced in young adults (aged 18-24 years).

**Meaning:**

These findings provide evidence of a growing mental health crisis in England and underscore an urgent need to address its cause and to adequately fund mental health services.

## Teaser

This survey study examines trends in psychological distress among adults in England from April 2020 to December 2022 and examines differences by key potential moderators.

## Introduction

Since 2020, England has undergone a period of substantial societal instability that may have contributed to worsening mental health. The COVID-19 pandemic brought with it an assortment of stressors, including fear of risk of infection, work and school closures, reduced social contact, financial strain, and uncertainty about the future.^1,2,3^ There has been a cost-of-living crisis in the UK since late 2021, whereby high rates of inflation have caused the cost of everyday essentials like groceries, energy, and household bills to increase faster than average household incomes.^4^ This has led to widespread industrial action since mid-2022, with unions across industries (including railways, the National Health Service, and education) striking for wage increases in line with inflation. There is also an ongoing health care crisis that has seen increased pressures on the National Health Service and across the health and social care sector, resulting in substantial delays for patients seeking emergency care.^5,6^ These national pressures have occurred in the context of other international emergencies, including the climate crisis and the war in Ukraine. Collectively, these circumstances may have increased levels of psychological distress in the population, particularly among groups with less disposable income or other vulnerabilities.^7^ It is important to understand whether, how, and among which groups there have been long-term shifts in population mental health burden, because this will have implications for service needs.^8^

Mental health problems are not experienced equally across population groups. Previous studies^9,10,11^ have identified a number of sociodemographic groups at greater risk of psychological distress, including younger adults, women, and people who are less socioeconomically advantaged (indicated by unemployment or lower income, education, or occupational status). Factors relating to family and household structure, including being single, living alone, and (less consistently) having children in the home have also been linked to poorer mental health,^11,12,13^ as have behaviors such as smoking and heavy alcohol consumption.^14,15,16,17^ Many of the groups who have historically had higher levels of distress have also experienced greater hardship during recent years, which may have compounded inequalities in mental health. For example, the early stages of the COVID-19 pandemic had greater social and financial impacts on younger adults, women, and those with lower incomes^18,19,20,21^ (although COVID-19 mortality rates were higher among older adults, men, and minoritized racial and ethnic groups^22^). The cost-of-living crisis has seen particularly high rates of food insecurity in households with children and those receiving state benefits.^23^ More recently, the health care crisis is likely to disproportionately affect groups who seek emergency care more frequently, including older adults, people from socioeconomically deprived areas, people who smoke, and those drinking at high-risk levels.^24,25,26^

Studies^27,28,29,30,31,32^ conducted early in the COVID-19 pandemic showed an acute increase in psychological distress and mental health symptoms in the UK population. Although these changes were observed across most population subgroups sampled, some studies reported greater deterioration in mental health among certain groups, including younger adults, women, those with greater socioeconomic disadvantage, and those with children in the home,^27,28,32,33,34,35^ the same groups experiencing greater social and financial impacts early in the pandemic.^18,19,20,21^ According to the nationally representative UK Household Longitudinal Study,^35^ the prevalence of clinically significant distress returned to prepandemic levels by September 2020, after restrictions on social interaction were eased. However, levels of distress increased again when the second wave of COVID-19 hit the UK in late 2020, with a particularly pronounced increase among those with school-aged children at home.^33^ How levels of psychological distress have continued to change in the context of subsequent waves of the pandemic, the cost-of-living crisis, the health care crisis, and other global issues—and the extent to which changes have differed between groups—is not known.

The Smoking and Alcohol Toolkit Study has been collecting data on psychological distress from a representative sample of adults in England each month since April 2020 (the first wave of data collected after the COVID-19 pandemic began to affect England in March 2020). It is, therefore, well placed to provide up-to-date descriptive information on levels of psychological distress and insight into trends over the entirety of this unstable period to date. This study used these data to estimate time trends in psychological distress and to explore differences by key potential moderators to identify high-risk groups. Specifically, we aimed to address 2 research questions. First, how has the prevalence of any and severe past-30-day psychological distress among adults in England changed since April 2020? Second, to what extent have changes in any and severe past-30-day psychological distress differed by age, gender, socioeconomic position (indexed by occupational social grade), presence of children in the household, smoking status, and drinking risk status?

## Methods

### Data Source and Study Sample

This survey study used data from the ongoing Smoking and Alcohol Toolkit Study, a monthly cross-sectional survey of a representative sample of adults (aged ≥18 years) living in households in England.^36,37^ The study uses a hybrid of random probability and simple quota sampling to select a new sample of approximately 1700 adults each month. Since April 2020, data have been collected via computer-assisted telephone interview. Comparisons with other national surveys indicate that key variables such as sociodemographic characteristics are nationally representative.^36^ For the present study, we analyzed trends in psychological distress in the period from April 2020 (the first data collected after the COVID-19 pandemic began to affect England) to December 2022 (the most recent data available at the time of analysis).

Ethical approval for the Smoking and Alcohol Toolkit Study was granted originally by the University College London ethics committee. The data are collected by Ipsos Mori and are anonymized when received by University College London. All participants provide verbal informed consent. The study conformed to American Association for Public Opinion Research (AAPOR) reporting guideline for survey research.

### Measures

Psychological distress was measured using the Kessler Psychological Distress Scale, which measures nonspecific psychological distress in the past month.^38,39^ It uses 6 questions: “During the past 30 days, about how often, if at all, did you feel (1) nervous, (2) hopeless, (3) restless or fidgety, (4) so depressed that nothing could cheer you up, (5) that everything was an effort, and (6) worthless?”

Responses were a 5-point scale, from 0 (none of the time) to 4 (all of the time) and were summed across items to produce a total score ranging from 0 to 24. We used established cutoffs to define severe (scores ≥13), moderate (scores 5-12) and no or minimal (scores <5) psychological distress.^40^ We analyzed any moderate or severe distress (scores ≥5) as our primary outcome referred to as any distress, and severe distress (scores ≥13) as a secondary outcome.

Age was categorized as 18 to 24, 25 to 34, 35 to 49, 50 to 64, or 65 or more years. Gender was self-reported as man, woman, or in another way and summarized descriptively. Those who identify in another way were excluded from the trend analyses by gender because of the low numbers.

Occupational social grade was categorized as AB (higher and intermediate managerial, administrative, and professional), C1 (supervisory, clerical and junior managerial, administrative, and professional), C2 (skilled manual workers), D (semiskilled and unskilled manual workers), and E (state pensioners, casual and lowest grade workers, and unemployed with state benefits only).^41^ The number of children in the household was self-reported and categorized as 0, 1, or 2 or more. Smoking status was self-reported and categorized as current, former, or never smoking.

Drinking risk status was assessed with the Alcohol Use Disorders Identification Test–Consumption. Scores of 5 or higher were defined as drinking at increasing or higher-risk levels (ie, levels that increase someone’s risk of harm), and scores less than 5 were defined as drinking at low-risk levels or not drinking.^42^

### Statistical Analysis

The analysis plan was preregistered on Open Science Framework.^43^ Data were analyzed in R statistical software version 4.2.1 (R Project for Statistical Computing). We excluded participants with missing data on our outcome of interest (psychological distress). Those with missing data on potential moderators were excluded on a per-analysis basis.

The Smoking and Alcohol Toolkit Study uses raking to weight the sample to the population in England on the dimensions of age, social grade, region, housing tenure, ethnicity, and working status within sex.^44^ This profile is determined each month by combining data from the 2011 UK Census, the Office for National Statistics midyear estimates, and the annual National Readership Survey.^36^ The following analyses used weighted data.

We used log-binomial regression to test the association of (1) any and (2) severe psychological distress with survey month. Survey month was modeled using restricted cubic splines with 5 knots, to allow associations with time to be flexible and nonlinear, while avoiding categorization.

To explore moderation by age, gender, social grade, presence of children in the household, smoking, and drinking risk status, we repeated the models including the interaction between the moderator of interest and survey month, thus allowing for time trends to differ across subgroups. Each of the interactions was tested in a separate model. Two-sided *P* < .05 was considered statistically significant. We used predicted estimates from our models to plot the prevalence of each outcome over the study period (overall and by moderating variables), alongside unadjusted (weighted) data, and reported prevalence ratios (PRs) for the change in prevalence across the whole time-series (December 2022 vs April 2020) alongside 95% CIs calculated using bootstrapping.

## Results

A total of 53 370 adults in England participated in the Smoking and Alcohol Toolkit Study between April 2020 and December 2022 (mean [SD], 1617 [42.1] participants per month). We excluded 1509 participants (2.8%) with missing data on distress, leaving an analytic sample of 51 861 participants (weighted mean [SD] age, 48.6 [18.5] years; 26 609 women [51.3%]). Compared with the analyzed sample, the group excluded for missing distress overrepresented people who were aged 18 to 24 years or 65 years and older, described their gender in another way, were from social grades C1 and E, currently smoked, drank at low-risk levels or not at all, and those who were surveyed in 2022 (eTable 1 in Supplement 1).

### Overall Estimates of Distress

Across the study period, 30.0% of adults reported any distress, and 6.2% reported severe distress (Table 1). Groups with notably higher prevalence of any and severe distress included younger adults, women and those who describe their gender in another way, those from less advantaged social grades, and those who currently smoke (Table 1). In addition, those with 1 child in the household had slightly higher prevalence of any distress than those with no children or 2 or more children, and those not drinking or drinking at low-risk levels had slightly higher prevalence of severe distress than those drinking at high-risk levels (Table 1). When we looked at differences by drinking risk status in more detail in an unplanned analysis, using the full spectrum of Alcohol Use Disorders Identification Test–Consumption scores, we saw the highest prevalence at either ends of the scale (ie, among those with the highest scores and not drinking; see eTable 2 in Supplement 1).

**Table 1.** Unadjusted Weighted Prevalence of Psychological Distress Among Adults in England: Data Aggregated Across the Study Period, April 2020-December 2022

Characteristic	Participants, No.^a^	Prevalence, % (95% CI)	
		Any distress	Severe distress
All adults	51 861	30.0 (29.5-30.4)	6.2 (6.0-6.4)
Age, y			
18-24	5581	53.6 (52.2-55.1)	14.0 (13.0-15.0)
25-34	7698	41.7 (40.6-42.9)	8.9 (8.2-9.6)
35-49	10 952	30.9 (30.0-31.8)	6.0 (5.5-6.5)
50-64	14 047	22.6 (21.8-23.3)	4.5 (4.1-4.9)
≥65	13 583	16.7 (16.0-17.4)	2.4 (2.1-2.6)
Gender			
Men	24 886	25.5 (24.9-26.2)	4.7 (4.4-5.0)
Women	26 609	33.9 (33.2-34.5)	7.4 (7.0-7.7)
Described in another way	307	59.6 (54.1-65.1)	25.7 (20.8-30.6)
Social grade			
AB (most advantaged)	12 464	23.3 (22.5-24.0)	2.7 (2.4-3.0)
C1	21 613	29.7 (29.1-30.3)	5.1 (4.8-5.5)
C2	8362	29.6 (28.6-30.6)	5.9 (5.4-6.4)
D	4370	36.0 (34.5-37.5)	9.2 (8.3-10.1)
E (least advantaged)	5052	42.5 (41.1-43.9)	16.0 (15.0-17.1)
Children in the household			
0	37 944	29.2 (28.7-29.7)	6.3 (6.0-6.5)
1	6220	33.4 (32.1-34.7)	6.5 (5.8-7.1)
≥2	7697	30.8 (29.7-31.9)	5.7 (5.1-6.3)
Smoking status			
Never smoking	29 957	26.5 (26.0-27.1)	4.3 (4.1-4.6)
Former smoking	13 646	28.3 (27.4-29.1)	5.5 (5.1-6.0)
Current smoking	7881	44.7 (43.5-45.9)	13.7 (12.9-14.5)
Drinking risk status			
Low-risk or nondrinking	33 528	30.1 (29.6-30.7)	6.3 (6.0-6.6)
High-risk drinking	16 802	29.4 (28.7-30.2)	5.7 (5.3-6.1)

^a^ Data reflect the unweighted sample size. Note that there were some missing data for gender (n = 59), smoking status (n = 377), and drinking risk status (n = 1531), so subgroups for these variables do not sum to the total sample size.

### Time Trends in Any Distress

**Figure 1.**
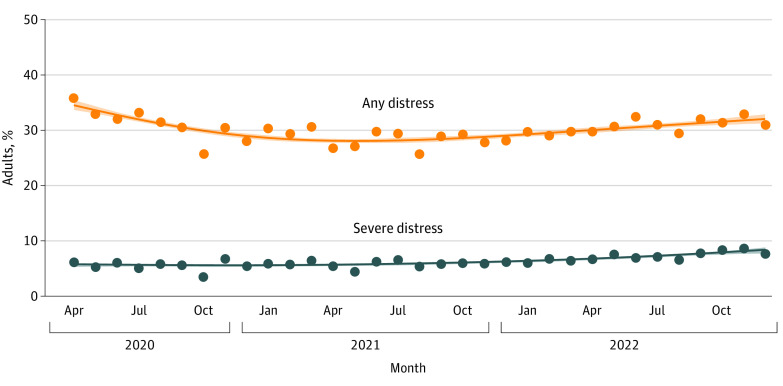
Time Trends in the Proportion of Adults in England Reporting Any Psychological Distress (Moderate or Severe) and Severe Psychological Distress, April 2020 to December 2022 Lines represent modeled weighted prevalence by survey month, modeled nonlinearly using restricted cubic splines (5 knots). Shaded bands represent SEs. Points represent observed weighted prevalence by month.

The proportion of adults reporting any distress decreased from 34.5% to 28.0% between April 2020 and May 2021, then increased to 32.0% by December 2022 (Figure 1), such that there was little overall change from the start to the end of the study period (PR, 0.93; 95% CI, 0.87-0.99) (Table 2). A significant overall decrease in any distress between April 2020 and December 2022 was observed among those aged 65 years and older (PR, 0.56; 95% CI, 0.46-0.68), women (PR, 0.86; 95% CI, 0.78-0.93), those from social grade C1 (PR, 0.86; 95% CI, 0.78-0.95), those with no children in the household (PR, 0.90; 95% CI, 0.82-0.97), those reporting never smoking (PR, 0.90; 95% CI, 0.81-0.996) or former smoking (PR, 0.85; 95% CI, 0.75-0.98), and those not drinking or drinking at low-risk levels (PR, 0.85; 95% CI, 0.78-0.93). No significant changes were observed in other subgroups (with PR estimates ranging from 0.88 to 1.08) (Table 2).

**Table 2.** Modeled Weighted Estimates of the Prevalence of Psychological Distress Among Adults in England in April 2020 and December 2022

Characteristic	Any distress (moderate or severe)			Severe distress		
	Prevalence, % (95% CI)		PR (95% CI)	Prevalence, % (95% CI)		PR (95% CI)
	April 2020^a^	December 2022		April 2020	December 2022	
All adults	34.5 (32.8-36.2)	32.0 (30.4-33.6)	0.93 (0.87-0.99)	5.7 (4.9-6.5)	8.3 (7.3-9.4)	1.46 (1.21-1.76)
Age, y						
18-24	55.5 (50.1-61.4)	58.7 (53.9-63.9)	1.06 (0.93-1.20)	11.1 (8.3-14.9)	20.2 (16.3-24.9)	1.81 (1.29-2.61)
25-34	44.4 (40.0-49.3)	42.9 (38.9-47.4)	0.97 (0.84-1.10)	7.9 (5.8-10.9)	11.7 (9.2-14.9)	1.48 (1.03-2.21)
35-49	34.6 (31.2-38.3)	35.8 (32.3-39.6)	1.03 (0.90-1.19)	6.1 (4.5-8.2)	7.2 (5.4-9.6)	1.19 (0.78-1.77)
50-64	27.3 (24.7-30.2)	24.0 (21.3-27.1)	0.88 (0.75-1.02)	3.8 (2.8-5.1)	6.2 (4.6-8.2)	1.62 (1.09-2.44)
≥65	25.2 (22.5-28.1)	14.1 (11.9-16.7)	0.56 (0.46-0.68)	3.2 (2.2-4.6)	2.5 (1.6-4.0)	0.79 (0.43-1.38)
Gender^b^						
Men	27.6 (25.4-30.0)	28.0 (25.8-30.3)	1.01 (0.91-1.14)	3.6 (2.8-4.7)	6.2 (5.0-7.7)	1.73 (1.28-2.43)
Women	41.2 (38.9-43.6)	35.2 (32.9-37.7)	0.86 (0.78-0.93)	7.6 (6.4-9.0)	9.7 (8.3-11.4)	1.28 (1.02-1.60)
Social grade						
AB (most advantaged)	27.5 (24.6-30.8)	24.1 (21.5-27.2)	0.88 (0.74-1.03)	2.3 (1.5-3.4)	3.4 (2.3-5.0)	1.52 (0.85-2.66)
C1	37.5 (34.9-40.2)	32.3 (30.0-34.8)	0.86 (0.78-0.95)	5.3 (4.2-6.7)	7.2 (5.9-8.6)	1.34 (1.01-1.82)
C2	30.5 (27.3-34.1)	29.3 (25.9-33.2)	0.96 (0.82-1.13)	4.3 (3.0-6.0)	7.3 (5.5-9.8)	1.71 (1.11-2.72)
D	39.6 (34.5-45.5)	39.4 (34.0-45.7)	1.00 (0.81-1.22)	9.0 (6.2-13.0)	14.4 (10.5-19.7)	1.60 (1.02-2.53)
E (least advantaged)	46.7 (41.0-53.1)	48.9 (43.6-54.9)	1.05 (0.88-1.25)	14.9 (11.2-19.7)	19.4 (15.4-24.5)	1.31 (0.93-1.92)
Children in the household						
0	33.9 (32.0-35.9)	30.4 (28.6-32.3)	0.90 (0.82-0.97)	5.7 (4.9-6.8)	8.7 (7.5-10.0)	1.51 (1.23-1.85)
1	38.8 (34.2-44.0)	37.5 (33.0-42.6)	0.97 (0.81-1.15)	5.6 (3.6-8.6)	7.5 (5.1-10.9)	1.35 (0.77-2.34)
≥2	33.3 (29.3-37.9)	34.4 (30.5-38.9)	1.03 (0.87-1.22)	5.4 (3.6-8.0)	7.0 (5.1-9.8)	1.32 (0.80-2.21)
Smoking status						
Never smoking	30.7 (28.7-32.9)	27.5 (25.6-29.7)	0.90 (0.81-1.00)	3.7 (3.0-4.6)	5.3 (4.3-6.5)	1.44 (1.07-1.95)
Former smoking	35.1 (32.1-38.4)	29.9 (27.0-33.2)	0.85 (0.75-0.98)	5.5 (4.1-7.3)	8.5 (6.6-10.9)	1.54 (1.06-2.20)
Current smoking	46.3 (42.0-51.1)	50.1 (45.9-54.8)	1.08 (0.95-1.23)	12.6 (9.9-16.0)	18.3 (15.1-22.1)	1.45 (1.10-2.02)
Drinking risk status						
Low-risk or nondrinking	36.0 (33.9-38.2)	30.5 (28.6-32.6)	0.85 (0.78-0.93)	6.2 (5.2-7.4)	7.3 (6.2-8.6)	1.17 (0.92-1.48)
High-risk drinking	31.8 (29.3-34.6)	34.1 (31.3-37.1)	1.07 (0.95-1.20)	4.5 (3.5-5.8)	9.7 (7.9-11.9)	2.16 (1.58-2.95)

Abbreviation: PR, prevalence ratio.

^a^ Data for April 2020 and December 2022 are weighted estimates of prevalence in these months (the first and last in the study period) from log-binomial regression with survey month modeled nonlinearly using restricted cubic splines (5 knots), allowing an interaction between survey month and the variable of interest (eg, between survey month and age for estimates by age).

^b^ Changes in prevalence of distress over time were not analyzed among those identifying their gender in another way owing to insufficient sample size.

**Figure 2.**
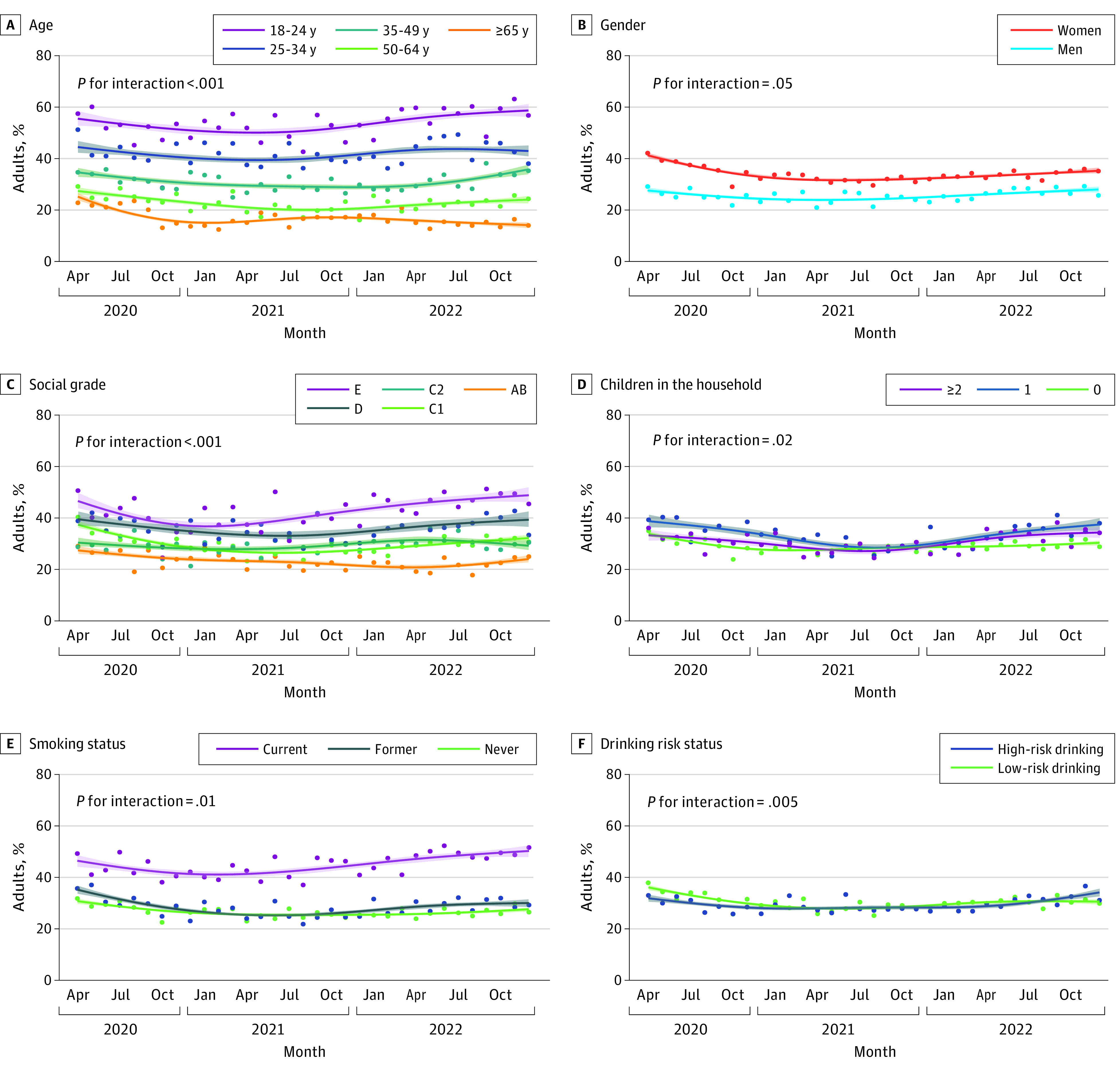
Time Trends in the Proportion of Adults in England Reporting Any Psychological Distress (Moderate or Severe), April 2020 to December 2022 Graphs show trends by age (A), gender (B), social grade (C), children in the household (D), smoking status (E), and drinking risk status (F). Lines represent modeled weighted prevalence by survey month, modeled nonlinearly using restricted cubic splines (5 knots). Shaded bands represent SEs. Points represent observed weighted prevalence by month.

However, trends in the prevalence of any distress within the study period differed significantly by all sociodemographic and behavioral characteristics (Figure 2). People aged 65 years and older showed different patterns of any distress compared with younger age groups—a more pronounced decline during 2020 and a decrease in any distress since late 2021—whereas the prevalence increased among younger adults (Figure 2A). There was a decrease in any distress during 2020 among women, but little change among men (Figure 2B). After an initial decrease in any distress across social grades (with the exception of C2, where the prevalence was stable), the subsequent increase occurred soonest among those in social grade E and latest among those in social grades AB (with C2 the only group to show a fall in 2022) (Figure 2C). People with 1 child in the household had the highest prevalence of any distress in April 2020 and a more pronounced decline through mid-2021; in addition, from late 2021, there was an increase in any distress among those with 1 or more children in the household, whereas the prevalence remained stable among those with no children in the household (Figure 2D). People who used to smoke showed a more pronounced decline in any distress during 2020 than those who currently or never smoked, and those who currently smoke showed a more pronounced increase since mid-2021 (Figure 2E). Those not drinking or drinking at low-risk levels showed a more pronounced decline in any distress during 2020 than those drinking at high-risk levels, and the latter group showed a more pronounced increase in any distress in 2022 (Figure 2F).

### Time Trends in Severe Distress

The proportion of adults reporting severe distress increased by 46% between April 2020 and December 2022 (PR, 1.46; 95% CI, 1.21-1.76) (Table 2), increasing steadily from 5.7% to 8.3% with no period of decline (Figure 1). An overall increase in severe distress between April 2020 and December 2022 was observed across all subgroups (with PR estimates ranging from 1.17 to 2.16) (Table 2), with the exception of those aged 65 years and older (PR, 0.79; 95% CI, 0.43-1.38). Of note, the proportion reporting severe distress increased by 9 percentage points among people younger than 25 years and by 5 percentage points among those from the most disadvantaged social grades (D and E) and current smokers.

**Figure 3.**
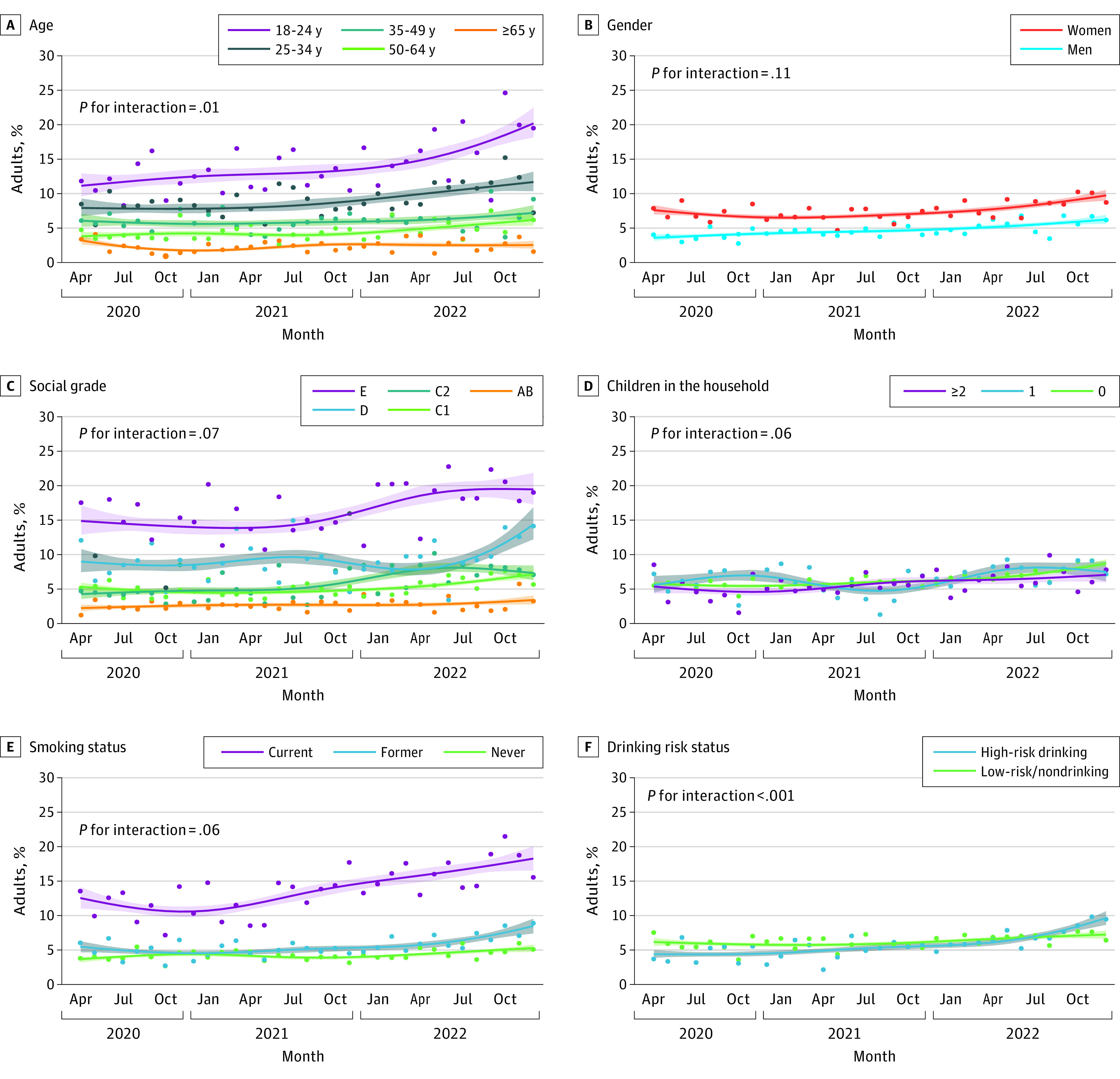
Time Trends in the Proportion of Adults in England Reporting Severe Psychological Distress, April 2020 to December 2022 Graphs show trends by age (A), gender (B), social grade (C), children in the household (D), smoking status (E), and drinking risk status (F). Lines represent modeled weighted prevalence by survey month, modeled nonlinearly using restricted cubic splines (5 knots). Shaded bands represent SEs. Points represent observed weighted prevalence by month.

Time trends in the prevalence of severe distress within the study period differed significantly by age (*P* for interaction = .01) and drinking risk status (*P* for interaction < .001). From late 2021, there was a sharp increase in severe distress among participants aged 18 to 24 years (from 13.6% in December 2021 to 20.2% in December 2022); smaller increases among those aged 25 to 34 years (from 9.9% in December 2021 to 11.7% in December 2022), those aged 35 to 49 years (from 6.1% in December 2021 to 7.2% in December 2022), and those aged 50 to 64 years (from 4.9% in December 2021 to 6.2% in December 2022); and no change among those aged 65 years and older (2.5% at both time points). Nearer the end of the study (April 2022 to December 2022), there was an increase in severe distress among those drinking at high-risk levels (from 6.2% to 9.7%), whereas levels remained stable among those not drinking or drinking at low-risk levels (at approximately 7%) (Figure 3F). Tests of interactions were inconclusive across other characteristics (with *P* for interaction ranging from .06 to .11) (Figure 3).

## Discussion

Between April 2020 and December 2022, there was little overall change in the proportion of adults in England reporting any distress (declining from 34.5% to 32.0%; PR, 0.93) but the proportion reporting severe distress increased by almost one-half from 5.7% to 8.3% (PR, 1.46). Within this period, the prevalence of any distress declined between April 2020 and May 2021 and then returned to slightly below baseline levels by December 2022, whereas the prevalence of severe distress increased consistently. It is important to note that the baseline assessment was conducted in April 2020, at the start of the COVID-19 pandemic, when people were experiencing substantial disruption to their daily lives and fear and anxiety about the pandemic (eg, they or loved ones contracting and becoming seriously ill from COVID-19) were at their highest.^45^ Other studies documented a notable increase in distress during the early months of the pandemic; for example, in the UK Household Longitudinal Study (a nationally representative panel study), the prevalence of clinically significant psychological distress (defined as a score of ≥4 of 12 on the General Health Questionnaire–12) increased from 21% before the pandemic (2019) to 30% in April 2020.^35^ This makes our findings even more concerning: the prevalence of any distress among adults in England at the end of the study (in December 2022) was only slightly lower than at the start of the pandemic, and the prevalence of severe distress was 46% higher.

There was a pronounced age gradient across the study period, with the lowest prevalence of both any and severe distress among the oldest age group (aged ≥65 years) and the highest prevalence among the youngest group (aged 18-24 years). The decline in any distress during the first year of the study was particularly pronounced among those aged 65 years and older. The participants aged 65 years and older were also the only subgroup we looked at not to show an increase in severe distress. Given that the health risks associated with COVID-19 were greatest for this age group,^22^ this group had the most reason to have comparatively high levels of distress at the start of this period. Over time, they benefited the most from the continued rollout of the vaccination program in terms of their reduction in risk of severe COVID-19 outcomes.^46^ Meanwhile, those aged 18 to 24 years showed the sharpest increase in severe distress, particularly in the last year of the study (increasing from 13.6% in December 2021 to 20.2% in December 2022). This younger group may have been more affected than older groups by recent stressors, such as the cost-of-living crisis (because they typically have less disposable income^47^), the climate crisis, and war in Ukraine. Regardless of the cause, the fact that 1 in 5 young adults reports severe distress is a cause for concern and warrants action by policy makers.

As has been observed in previous studies,^35,48^ women reported higher levels of distress than men. There was a decrease in any distress in 2020 among women but little change among men, which narrowed the gender gap but did not close it entirely. This result may reflect easing of the childcare burden during the COVID-19 pandemic, which disproportionately fell on women.^19,20^ The prevalence of distress was also very high among those who described their gender in another way—substantially greater than those identifying as women or men—across the whole period, but we were unable to analyze trends in this group owing to the small sample size.

Occupational social grade was negatively associated with distress, consistent with previous literature documenting a substantial socioeconomic gradient in health, including mental health and well-being.^49,50^ The increases in prevalence of any and severe distress we observed occurred soonest among social grade E (the most disadvantaged group). This group started from a high baseline and experienced a large 5 percentage point increase in severe distress. This may be explained by this group being hit earlier by the cost-of-living crisis, because they had less disposable income to absorb increasing costs of household essentials. A survey^51^ conducted in July 2022 found that almost one-half (42%) of people living in the most deprived quintile of areas in England had cut back on food and essentials since the cost-of-living crisis began, compared with 27% in the least deprived quintile.

Patterns of distress varied by the number of children in the household. Those with 1 child had the highest prevalence of any distress at the start of the study period in April 2020 than those with none or multiple children. It is possible that this may be because 2 or more children provided company for each other, meaning parents were less worried about a lack of interaction with peers or the need to provide entertainment during lockdown. There was an increase in any distress among those with 1 or more children since late 2021, which may be linked to the additional strain having children puts on household budgets^52^ in the context of the cost-of-living crisis. Parents may also be concerned about their children’s futures, for example due to impending climate hazards.

The prevalence of distress was elevated among those who currently smoked. It is a common misconception that smoking helps to relieve stress,^53^ when in fact levels of distress are typically higher among people who smoke and decrease when people quit.^54^ The most pronounced decline in any distress in the early part of the study period was observed among people who reported former smoking. This was likely confounded with age, since former smokers are, on average, older than never and current smokers.^55^ Similarly, the increase in any distress in the later part of the study was more pronounced among those who currently smoked, which is likely confounded with social grade as smoking is much more common among socioeconomically disadvantaged groups.^56^

Levels of distress were similar between those drinking at high-risk levels and those not drinking or drinking at low-risk levels. This was explained by relatively higher prevalence of distress among people not drinking, which may be caused by those in poor health (and thereby greater distress) abstaining from drinking^57^ (levels of distress were higher among those drinking at high-risk than those reporting low-risk levels of consumption). There was a more pronounced increase in distress near the end of the study period among those drinking at high-risk levels. It is possible that people experiencing distress related to the cost-of-living crisis or other stressors around this period were using alcohol as a coping strategy.^58^ The high burden of mental health problems in England is not necessarily a new concern,^59^ but the COVID-19 pandemic, cost-of-living crisis, and other stressors appear to have exacerbated the problem and caused existing inequalities in mental health to deepen. Groups with particularly high prevalence of distress include young adults, women, those who describe their gender in another way, people from more disadvantaged social grades, and people who smoke. Mitigating and managing these mental health needs requires adequately resourced services.^60^

### Limitations

This study had several limitations. Because it was a household survey, people too unwell to participate or those living in institutions were excluded, so the findings may underestimate levels of distress by excluding those experiencing severe mental health problems. In addition, although the sample was representative, the small proportion (2.8%) of participants who did not respond to the measure of distress tended to belong to groups with higher levels of distress (eg, those aged 18-24 years or describing their gender in another way), which may bias estimates of prevalence downward, as has been noted in previous studies.^61^ The numbers of participants reporting severe distress were small, limiting statistical power to detect significant differences in time trends between subgroups. Data on psychological distress were not collected in the survey before April 2020, so we were unable to draw comparisons with the prepandemic period. In addition, the survey did not capture other variables that may have been associated with changes in distress since April 2020, such as ethnicity, family circumstances (eg, living alone and marital status), economic factors (eg, job loss and food insecurity), or health status (eg, disability and diagnosed conditions). Nonetheless, it provides a comprehensive summary of trends in distress over this period. Although we have speculated on the potential causes of the patterns of distress we have observed across population groups, further research (eg, qualitative) is required to provide deeper insight into the factors that have caused a surge in the proportion of adults experiencing distress, how they differ between population groups, and how to reduce their impact.

## Conclusions

In this survey study of adults in England, the proportion reporting any psychological distress was similar in December 2022 to that in April 2020 (an extremely difficult and uncertain moment of the COVID-19 pandemic), and the proportion reporting severe distress was 46% higher. This burden has been compounded by a particularly sharp increase in severe distress since 2021 among young adults. Because not all people experiencing distress or other symptoms of mental health problems will seek treatment, continued monitoring outside of clinical populations is important for understanding the scale of the mental health crisis in England and introducing measures to address it.

## Notes

Supplement 1. **eTable 1.** Weighted Characteristics of the Analysed Sample Compared With Participants Excluded on the Basis of Missing Distress Data **eTable 2.** Weighted Prevalence of Psychological Distress Among Adults in England, by AUDIT-C Score and 3-Level Drinking Risk Status: Data Aggregated Across the Study Period (April 2020-December 2022)

Supplement 2.
Data Sharing Statement
