# Supplementary material for: Trends in Psychological Distress Among Adults in England, 2020-2022
Source: JAMA Netw Open. 2023 Jul 6;6(7):e2321959. doi: 10.1001/jamanetworkopen.2023.21959 (PMC10326642; doi:10.1001/jamanetworkopen.2023.21959)
Supplement: Supplement 1. — eTable 1. Weighted Characteristics of the Analysed Sample Compared With Participants Excluded on the Basis of Missing Distress Data eTable 2. Weighted Prevalence of Psychological Distress Among Adults in England, by AUDIT-C Score and 3-Level Drinking Risk Status: Data Aggregated Across the Study Period (April 2020-December 2022) [file jamanetwopen-e2321959-s001.pdf]

Supplemental Online Content

Jackson SE, Brown J, Shahab L, McNeill A, Munafò MR, Brose L. Trends in psychological distress among adults in England, 2020-2022. *JAMA Netw Open*. 2023;6(7):e2321959. doi:10.1001/jamanetworkopen.2023.21959

eTable 1. Weighted Characteristics of the Analysed Sample Compared With Participants Excluded on the Basis of Missing Distress Data
eTable 2. Weighted Prevalence of Psychological Distress Among Adults in England, by AUDIT-C Score and 3-Level Drinking Risk Status: Data Aggregated Across the Study Period (April 2020-December 2022)

This supplemental material has been provided by the authors to give readers additional information about their work.

© 2023 Jackson SE et al. *JAMA Network Open.*

eTable 1. Weighted Characteristics of the Analysed Sample Compared With Participants Excluded on the Basis of Missing Distress Data

	**Analysed sample, %** (N = 51,861)	**Missing distress data, %** (N = 1,509)	***p***
Age			<0.001
18-24	11.4 (11.1-11.7)	13.4 (11.6-15.4)	-
25-34	17.3 (16.9-17.7)	19.5 (17.3-21.8)	-
35-49	22.9 (22.5-23.3)	22.4 (20.1-24.9)	-
50-64	25.3 (24.9-25.7)	19.3 (17.2-21.5)	-
≥65	23.1 (22.8-23.5)	25.5 (23.3-27.8)	-
Gender			<0.001
Men	48.7 (48.2-49.2)	49.2 (46.4-51.9)	-
Women	50.7 (50.3-51.2)	49.2 (46.5-51.9)	-
In another way	0.6 (0.5-0.7)	1.6 (1.1-2.4)	-
Missing, N	*59*	*6*	-
Social grade			<0.001
AB	27.0 (26.6-27.4)	18.0 (16.0-20.3)	-
C1	29.3 (28.9-29.6)	33.1 (30.8-35.5)	-
C2	20.7 (20.3-21.1)	19.0 (16.7-21.4)	-
D	14.2 (13.8-14.6)	15.9 (13.7-18.5)	-
E	8.9 (8.7-9.2)	14.0 (12.3-15.9)	-
Children in the household			0.45
0	71.0 (70.6-71.5)	72.3 (69.7-74.8)	-
1	12.8 (12.4-13.1)	11.5 (9.9-13.5)	-
≥2	16.2 (15.9-16.6)	16.1 (14.2-18.3)	-
Smoking status			0.001
Never smoking	57.7 (57.2-58.2)	57.1 (54.3-59.8)	-
Former smoking	25.7 (25.3-26.1)	22.8 (20.6-25.1)	-
Current smoking	16.6 (16.2-16.9)	20.2 (18.0-22.5)	-
Missing, N	*377*	*40*	-
Drinking risk status			<0.001
Low-risk/non-drinking	66.7 (66.3-67.2)	77.8 (75.3-80.2)	-
High-risk drinking	33.3 (32.8-33.7)	22.2 (19.8-24.7)	-
Missing, N	*1,531*	*190*	-
Year of survey			<0.001
2020	28.0 (27.5-28.4)	18.1 (16.1-20.3)	-
2021	36.6 (36.1-37.0)	37.7 (35.1-40.4)	-
2022	35.5 (35.0-35.9)	44.2 (41.5-46.9)	-

Data shown are weighted proportions. Missing data are shown as unweighted *n*s.

© 2023 Jackson SE et al. *JAMA Network Open.*

eTable 2. Weighted Prevalence of Psychological Distress Among Adults in England, by AUDIT-C Score and 3-Level Drinking Risk Status: Data Aggregated Across the Study Period (April 2020-December 2022)

		Prevalence, % (95% CI)	
	N [1]	**Any distress**	**Severe distress**
**AUDIT-C score**			
0	11,544	34.4 (33.4-35.3)	8.8 (8.2-9.3)
1	4,676	30.4 (29.0-31.8)	6.8 (6.0-7.7)
2	4,828	29.6 (28.2-31.0)	5.1 (4.4-5.8)
3	5,740	26.5 (25.2-27.7)	3.9 (3.3-4.4)
4	6,740	25.9 (24.7-27.0)	4.4 (3.8-4.9)
5	4,874	27.0 (25.6-28.3)	4.4 (3.7-5.0)
6	3,800	30.8 (29.1-32.4)	5.3 (4.5-6.2)
7	2,604	29.5 (27.6-31.4)	5.3 (4.4-6.3)
8	2,081	30.4 (28.2-32.6)	6.5 (5.4-7.7)
9	1,571	27.5 (25.1-29.9)	5.6 (4.3-6.8)
10	1,075	29.8 (26.8-32.8)	7.6 (5.7-9.4)
11	504	36.3 (31.7-40.9)	11.0 (8.0-13.9)
12	293	42.9 (36.8-49.1)	15.7 (11.1-20.3)
**Drinking risk status**			
Non-drinking	11,544	34.4 (33.4-35.3)	8.8 (8.2-9.3)
Low-risk drinking	21,984	27.8 (27.2-28.5)	4.9 (4.6-5.3)
High-risk drinking	16,802	29.4 (28.7-30.2)	5.7 (5.3-6.1)

CI, confidence interval.

[1] Unweighted sample size: note there were some missing data for AUDIT-C (*n*=1,531) so subgroups do not sum to the total sample size.

Note: AUDIT-C scores of 0 were considered non-drinking, 1-5 low-risk drinking and ≥5 high-risk drinking.

© 2023 Jackson SE et al. *JAMA Network Open.*
