# Supplementary material for: Trends in Psychological Distress Among Adults in England, 2020-2022
Source: JAMA Netw Open. 2023 Jul 6;6(7):e2321959. doi: 10.1001/jamanetworkopen.2023.21959 (PMC10326642; doi:10.1001/jamanetworkopen.2023.21959)
Supplement: Supplement 2. — Data Sharing Statement [file jamanetwopen-e2321959-s002.pdf]

Data Sharing Statement

Jackson. Trends in Psychological Distress Among Adults in England, 2020-2022. *JAMA Netw Open*. Published July 06, 2023. doi:10.1001/jamanetworkopen.2023.21959

Data
Data available: No

Additional Information
Explanation for why data not available: Data will be available from the corresponding author on reasonable request
